# Optimal Energy Consumption Tasks Scheduling Strategy for Multi-Radio WSNs

**DOI:** 10.3390/s20030881

**Published:** 2020-02-07

**Authors:** Qiao Yan, Wei Peng, Guiqing Zhang

**Affiliations:** 1 School of Information and Electrical Engineering, Shandong Jianzhu University, Jinan 250101, China; yanqiao2016@163.com (Q.Y.); qqzhang@sdjzu.edu.cn (G.Z.); 2 The Key Laboratory of Intelligent Buildings Technology of Shandong Province, Shandong Jianzhu University, Jinan 250101, China

**Keywords:** energy consumption, task scheduling, multi-radio, wireless sensor networks, particle swarm optimization

## Abstract

Multi-radio technology is regarded as a promising way to improve the performance of Wireless Sensor Networks (WSNs) and has attracted much attention of researchers. It is very important to reduce energy consumption and to prolong the lifetime of Multi-Radio WSNs (MR-WSNs), since the node is generally battery-operated in MR-WSN environments. In this paper, two typical types of energy consumption process, the transmitting energy consumption and idle listening energy consumption, are analyzed firstly. Based on the above analysis, the energy consumption model of multi-radio nodes is built, and then it is considered as the optimization objective for the minimization energy consumption of multi-radio nodes. Furthermore, the heuristic optimal energy consumption task scheduling strategy based on the Particle Swarm Optimization (PSO) algorithm is proposed, and then the detailed steps of the proposed strategy are presented. Finally, the effectiveness and performance of the proposed strategy are evaluated through practical experiments and simulations. Evaluation results show that the proposed strategy outperforms some other algorithms, in terms of energy consumption, network lifetime, and tasks extensibility.

## 1. Introduction

Wireless Sensor Networks (WSNs) have been widely applied for healthcare/environment monitoring, smart gird, industrial monitoring, etc. [1,2,3,4]. Especially with the development of the Internet of Things (IoT), it has emerged as a ubiquitous part of modern communication networks. The rapid developments of WSNs and potential applications have increasing demands of network performance, including higher throughput, lower latency, longer lifetime and lower energy consumption [5,6,7]. For example, in the time-sensitive applications, including industrial monitoring, healthcare monitoring, etc., the higher latency may lead to serious industrial accident and/or to cause a patient to miss the chance of emergency aid. In the environment monitoring, longer network lifetime and lower energy consumption are the most important factors for achieving long duration continuous monitoring.

Normally, the base-station and remote nodes are equipped with a single radio transceiver. However, the single radio transceiver limits the network performances, such as the throughput, lifetime, and bandwidth, etc. In order to solve the above-mentioned issues, multi-radio technology was presented to sharply improve the network performance by taking the advantage of high capacity provided by the multi-radio concurrent communication ability. Therefore, the base station and remote nodes can provide a multi-radio transceiver and can construct Multi-Radio Wireless Sensor Networks (MR-WSNs). Multi-radio technology has successfully aroused many researcher’s interest and made them pay more attention to it [8,9,10]. Energy consumption which is one of the key research problems in the WSN fields is particularly prominent in the MR-WSN environment, especially when the multi-radio nodes operate by un-chargeable battery. Hence, it is significant to research how to reduce the energy consumption in MR-WSNs for prolonging the network lifetime. The advanced multi-radio task scheduling scheme will directly affect the energy consumption performance, and then further improve the network lifetime. Inspired by the different evolution algorithm, this paper researches the optimal energy consumption task scheduling strategy for the MR-WSN fields in order to reduce the energy consumption and to prolong the network lifetime. In this paper, the energy consumption of the multi radio is firstly converted into a multi-task scheduling problem, and then the analysis of energy consumption under two types of task status is detailed, including transmission state and idle listening state. Based on that analysis, the energy consumption model under those two statuses is built and is further taken as an optimal objective function of the multi-task scheduling problem in MR-WSNs. In order to solve the optimal energy consumption task scheduling problem, the multi-task scheduling strategy is designed based on the Particle Swarm Optimization (PSO) algorithm and its detailed steps are provided. Furthermore, both experimental and simulation results are given. Those results clearly indicate that the proposed strategy outperforms other schemes in terms of energy consumption, network lifetime, etc. Finally, the task extensibility of the proposed scheme is tested in order to obtain the best performance with the optimal number of tasks. The contributions of this paper can be summarized as follows.

- Analyzes the multi-task (typically the transmission task and idle listening task) scheduling process of multi-radio nodes. In our analysis, the tasks are divided into two sets, i.e., the waiting scheduling task set and the scheduled task set. In detail, the scheduled task set is further distinguished as transmitted packets and un-transmitted packets in order to obtain the accurate energy consumption model from the task scheduling perspective.
- Converts the energy consumption problem into a multi-task scheduling process and builds the accurate energy consumption model of MR-WSNs under the above two types of task status; obtains the optimization objective function and the corresponding constrained conditions of energy consumption for MR-WSNs. Hence, the energy consumption problem finally turns into an optimization problem with constraints.
- Introduces the PSO algorithm to solve the constrained optimization problem and designs the optimal energy consumption task scheduling strategy. Then provides the detailed key steps and pseudo-code of the optimization process. Meanwhile, the performance is also evaluated by experiments and simulations. Furthermore, the task extensibility of the proposed strategy is tested in order to confirm the optimal performance and the best number of tasks.

The remainder of this paper is organized as follows. Section 2 highlights the related work. In Section 3, detailed analysis of the energy dissipation process is provided and the model of task scheduling energy consumption is given. In Section 4, the constrained optimization objective function of energy consumption is built, and the energy-efficient task scheduling scheme using a PSO algorithm is proposed and the detailed steps are also presented. In Section 5, both experiments and simulations are deployed to evaluate the performance of the proposed task scheduling scheme. Finally, the conclusion is presented.

## 2. Related Work

It is critical to research how to reduce the energy consumption for MR-WSNs, since MR-WSNs consist of low power devices that are generally equipped with un-rechargeable/un-replaceable batteries and are generally distributed in geographically isolated areas. Therefore, many researchers have paid more attention to this topic in recent years. In [11], the authors proposed an optimal routing method based on convex theory for multi-radio multi-channel wireless mesh networks. Li et al. [12] formulated the routing, scheduling, channel assignment and power control problem in multi-power-level multi-radio WSNs as an integer linear programming problem, and then designed a distributed routing protocol which could significantly reduce energy consumption. In order to meet the data-intensive requirements of the structural health monitoring (SHM) field, a novel binary hybrid meta-heuristic algorithm was proposed for multi-radio multi-channel multi-power WSNs [13,14]. Elshrkawey et al. [15] improved the cluster head selection method and then proposed an enhanced schedule approach based on the modified Time Division Multiple Access (TDMA) scheme in order to minimize the energy dissipation during network communications. In [16], Zhang et al. proposed an optimal model of energy consumption for nodes, in which the residual energy and energy consumption of communication are considered. Moreover, they applied the Nash Equilibrium of cooperative game theory to solve the optimal solutions of the built model. Emad S. Hassan et al. [17] proposed a new self-healing scheme based on a single flow-controlled mobility within a cluster to make a trade-off between self-healing and energy consumption in mobile Unattended Wireless Sensor Networks. Tamandani et al. [18] proposed a two-step fuzzy logic system to enhance the cluster heads selection, further reduce the energy consumption, and prolong the lifetime of the WSNs. Roselin et al. [19] proposed a novel energy efficient connected coverage scheduling to maximize the lifetime of the WSNs. Zhang et al. [20] presented a novel secret confusion-based energy saving and privacy preserving data aggregation algorithm to reduce data traffic and energy consumption in WSNs. Peng et al. [21,22] applied the interval type-2 fuzzy logic system (IT2FLS) to achieve the multi-radio resource scheduling problem. Moreover, in [23], Peng et al. took the advantage of dealing with uncertainties of IT2FLS to handle the transmission power control problem and gave the power allocation strategy for maximization the lifetime of WSNs. In [24], Dhami et al. proposed an energy efficient genetic algorithm based approach with the concept of virtual grid based dynamic routes adjustment which enhanced the overall performance of WSNs.

The complex energy consumption optimization problems in WSNs, especially when the objective function contains many variables, have been proven to be a non-deterministic polynomial-complete problem (NP-complete). Hence, several heuristic algorithms, such as the differential evolution (DE) algorithm [25,26,27,28,29], the genetic algorithm (GA) [30,31,32,33], PSO [34,35,36,37,38], etc., are usually preferred to search the optimal result.

In order to maximize the coverage and to prolong network lifetime, the DE algorithm and the PSO algorithm were, separately, proposed to solve clustering the problem in WSNs [25,26]. In [27], the DE algorithm was presented for the situation in which the communication range value was unknown in order to deal with the non-convex optimization problem. In [28], the authors applied the multi-objective DE algorithm to jointly optimize the sensor distribution over diverse area shapes, increase the coverage area and reduce the network energy at the same time. For the path optimization problem in WSN, the DE algorithm was used to solve the minimum energy consumption and obtain the optimal routing path [29].

Moreover, the GA based modified LEACH algorithm was proposed for the energy harvesting WSNs (EH-WSNs) in order to minimize energy consumption and to prolong the network lifetime of the EH-WSNs [30]. Similarly, a self-organizing network clustering method based on GA was proposed for searching an optimal network structure, and further for extending the network lifetime [31]. In [32], the authors proposed a distributed GA to solve the energy efficient coverage problem in WSNs. In [33], the authors proposed GA based approaches for clustering and routing in WSNs in order to prolong the lifetime of a sensor and to increase the quality of service.

Inspired by the social behaviour of birds, PSO has became one of the most popular optimization algorithms and has been widely applied to solve complex optimization problems. A hybrid approach based on combining PSO with random transition moves was proposed to create the maximum possible number of disjoint sets in order to maximize the lifetime of WSNs [34]. In [35], the authors applied the PSO algorithm to decrease the location error and improve the location accuracy. In [36], Wang et al. presented an energy center searching method using the PSO algorithm to avoid energy holes near the cluster cased by the heavy burden of forwarding. In order to solve the resource allocation and energy consumption problem in multi-agent clustering WSNs, a hierarchical resource allocation strategy using the adaptive PSO algorithm was presented to address the issue of resource allocation in these types of networks [37]. In [38], the authors presented three different energy efficient routing strategies using the PSO algorithm; one for maximizing the energy of the node with the lowest energy, one for maximizing the overall WSN energy, and one for maximizing the energy of the worst performing node.

In summary, on one hand, it is great that the energy consumption optimization in WSNs has been researched and has been proven to be an NP-Complete problem. On the other hand, a heuristic algorithm has been applied to solve those optimization problems, including routing, cluster head selection, and energy consumption optimizaiton, etc. However, to our knowledge, such a heuristic algorithm is rarely applied for achieving energy save for MR-WSNs. Therefore, this paper introduces the PSO algorithm to the MR-WSNs and presents an optimal energy consumption multi-task scheduling strategy in order to reduce energy consumption and prolong the network lifetime for the MR-WSNs.

## 3. Multi-Tasks Schedule Formulation and Energy Consumption Model

In this section, the multi-task schedule process in the MR-WSN environment is formulated firstly. Then two types of energy consumption processes, the transmitting energy consumption and idle listening energy consumption, are detailed and analyzed separately. Then the accurate energy consumption model of the multi-radio node is obtained by combining those two processes together.

### 3.1. Multi-Task Schedule Description

For convenience, we firstly list all the related acronyms and parameters, as shown in Table 1.

**Figure 1:**
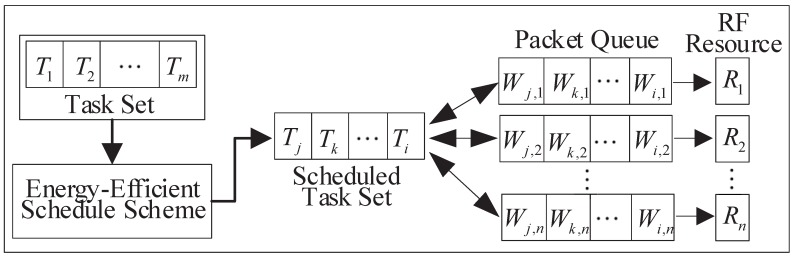
Task scheduling process.

For any one multi-radio node, suppose there are *n* half-duplex RF transceivers equipped on the node and the RF resource set is denoted by $R=\left\{R_{1},R_{2},\cdots ,R_{n}\right\}$. At any time, the number of tasks waiting to be scheduled is *m* and is denoted by $T=\left\{T_{1},T_{2},\cdots ,T_{m}\right\}$, the number of idle RF resources is γ, normally m≥γ. To make the description convenient, assume the number of packets which are transmitted by each task is *l*. The *l* packets of task $T_{i}$ are denoted by $W=\left\{W_{i,1},W_{i,2},\cdots ,W_{i,l}\right\}$. As shown in Figure 1, the energy efficient task scheduling strategy will quickly allocate the tasks according to the current RF resource and the corresponding packet queue in order to reduce the energy consumption and data latency.

### 3.2. Energy Consumption Model

For the *i*-th multi-radio node $N_{i}$, the *h* packets allocated to the *j*-th RF transceiver $R_{j}$ is denoted as $w_{i}^{R\left(j\right)}=\left\{w_{i,k},w_{i,k+1},\cdots ,w_{i,k+h}\right\}$. The required time for transmitting the *q*-th packet $w_{i,q}$ is denoted as $t_{i,q}$. Thus, the total required time for sending $w_{i}^{R\left(j\right)}=\left\{w_{i,k},w_{i,k+1},\cdots ,w_{i,k+h}\right\}$ can be obtained as follows

(1) $$t_{j}=\overset{q=k+h}{\sum_{q=k}}t_{i,q}$$

where $t_{j}$ is the total required time of $R_{j}$ for transmitting the *h* packets $w_{i}^{R\left(j\right)}=\left\{w_{i,k},w_{i,k+1},\cdots ,w_{i,k+h}\right\}$.

Therefore, we can obtain the time of each RF transceiver required based on Equation (1) and denote them as $t_{1},t_{2},\cdots ,t_{n}$. The maximum value of $t_{j}$ is denoted as $t_{\max}$ and

(2) $$t_{\max}=\max\left(t_{1},t_{2},\cdots ,t_{n}\right)$$

Energy consumption for transmitting $w_{i,q}$ is denoted as $S_{i,q}$, and

(3) $$S_{i,q}=P_{trans,j}*t_{i,q}$$

where $P_{trans,j}$ is the transmission power of $R_{j}$, $t_{i,q}$ is the required time for transmitting the packet $w_{i,q}$.

The total energy consumption for transmitting $w_{i}^{R\left(j\right)}=\left\{w_{i,k},w_{i,k+1},\cdots ,w_{i,k+h}\right\}$ is denoted as $S_{j}$, and

(4) $$\begin{array}{ccc} S_{j} & = & \underset{q=k}{\sum^{q=k+h}}S_{i,q}\\ & = & \underset{q=k}{\sum^{q=k+h}}P_{trans,j}*t_{i,q} \end{array}$$

The status of $R_{j}$ is fixed to idle listening after $w_{i}^{R\left(j\right)}=\left\{w_{i,k},w_{i,k+1},\cdots ,w_{i,k+h}\right\}$ are transmitted completed until the last packet that belongs to the task $T_{i}$ and is allocated to the other RF transceivers is transmitted completed. Therefore, the energy consumption in idle listening state is denoted as $I_{j}$ and

(5) $$I_{j}=p_{idle,j}*\left(t_{\max}-t_{j}\right)$$

where $p_{idle,j}$ is the idle listening power of $R_{j}$.

Thus, the total energy consumption of $R_{j}$ is denoted as $E_{j}$ and

(6) $$\begin{array}{ccc} E_{j} & = & S_{j}+I_{j}\\ & = & \underset{q=k}{\sum^{q=k+h}}P_{trans,j}*t_{i,q}+p_{idle,j}*\left(t_{\max}-t_{j}\right)\\ & = & \underset{q=k}{\sum^{q=k+h}}\left(P_{trans,j}-p_{idle,j}\right)*t_{i,q}+p_{idle,j}*t_{\max} \end{array}$$

Hence, the total energy consumption of $N_{i}$ is denoted as *E* and

(7) $$\begin{array}{ccc} E & = & \underset{j=1}{\sum^{j=n}}E_{j}\\ & = & \underset{j=1}{\sum^{j=n}}\underset{q=k}{\sum^{q=k+h}}\left(P_{trans,j}-p_{idle,j}\right)*t_{i,q}+\underset{j=1}{\sum^{j=n}}p_{idle,j}*t_{\max} \end{array}$$

Equation (7) reflects that the total energy consumption mainly consists of the transmission energy consumption and the idle listening energy consumption. The first part is related to the transmission power, the idle listening power and the required time. The second part is related to the idle listening power and the maximum transmission time. Suppose the transmission power and the idle listening power are constant, the first part of the total energy consumption is linearly correlated with the transmission time. Similarly, the second part is linearly correlated with the maximum transmission time.

## 4. Optimization Energy-Consumption Task Scheduling Strategy

In this section, the objective function to minimize is built firstly. Then the optimization energy consumption task scheduling scheme using different evolution algorithms is proposed. Moreover, the key steps of the proposed scheme are presented. The complexity of the proposed algorithm is analyzed at the end.

### 4.1. Multi-Objective Optimization Model

In application, the number of RF transceivers of the multi-radio node equipped is variable from 1 to 8. The range of transmission power and idle listening power are 0 dB to 17 dB and –10 dB to 0 dB, respectively. The maximum number of packets belonging to the same task and allocated to the same RF transceiver is 128. Hence, the objective function of optimization energy consumption task scheduling for MR-WSNs can be obtained.

(8) $$\min\left(\sum_{j=1}^{j=n}\sum_{q=k}^{q=k+h}\delta_{j}*t_{i,q}+\sum_{j=1}^{n}\gamma_{j}*t_{\max}\right)$$

(9) $$s.t.\left\{\begin{array}{c} \delta_{j}=P_{trans,j}-p_{idle,j}\\ \gamma_{j}=p_{idle,j}\\ t_{\max}\geq t_{j}\left(j=1,2,\ldots ,n\right) \end{array}\right.$$

Constraints.

(10) $$\left\{\begin{array}{c} 0dB\leq P_{trans,j}\leq 17dB\\ -10dB\leq p_{idle,j}\leq 0dB\\ 1\leq n\leq 8\\ 0\leq h\leq 128 \end{array}\right.$$

### 4.2. Design of the Strategy

In PSO, each solution is regarded as a particle which consists of a position and velocity. The velocity of each particle calculates iteratively according to its own experience and the global particles. Moreover, the particle updates its position based on the current velocity. The above process continues iteratively until it achieves an optimal (or near-optimal) solution or reaches the maximum number of iterations.

In our designed strategy, the number of particles is assumed to be equal to the number of tasks to be scheduled, and the search space is assumed to be D-dimensional. Thus, for the *i*-th particle, the position and the velocity can be, respectively, denoted $X_{i}=\left(X_{i1,}X_{i2,}\ldots ,X_{iD}\right)$ as $V_{i}=\left(V_{i1,}V_{i2,}\ldots ,V_{iD}\right)$. The iterative process of the position and velocity can be expressed as follows.

(11) $$V_{id}^{t+1}=\omega \times V_{id}^{t}+c_{1}\times rand\left(\right)\times \left(P_{id,best}^{t}-X_{id}^{t}\right)+c_{2}\times rand\left(\right)\times \left(G_{id,best}^{t}-X_{id}^{t}\right)$$

(12) $$X_{id}^{t+1}=X_{id}^{t}+V_{id}^{t+1}$$

where ω is called the inertia factor and ω≥0. $V_{id}^{t}$ and $V_{id}^{t+1}$ are the velocity of the *i*-th particle at the *t*-th (current velocity) and (t+1)-th, respectively. Similarly, the $X_{id}^{t}$ and $X_{id}^{t+1}$ are the position of the *i*-th particle at the *t*-th (current position) and t+1-th, respectively. $c_{1}$ and $c_{2}$ are the learning factors, normally fixed $c_{1}=c_{2}=2$. rand() is used to generate a random within [0,1]. $P_{id,best}^{t}$ and $G_{id,best}^{t}$ represent the current local optimal position of the *i*-th particle and the global optimal position of the particles.

The steps of the PSO used for the proposed optimization energy consumption task scheduling strategy are as follows.

**Step 1:** Randomly initialize every particle, including the velocity and position;

**Step 2:** Evaluate the objective function value for each particle and find the current local best position $P_{id,best}^{t}$. Then determine the global optimal position of the particles $G_{id,best}^{t}$;

**Step 3:** Update the velocity and position of each particle according to Equations (11) and (12), separately. Then update the current local best position $P_{id,best}^{t}$ and the global optimal position of the particles $G_{id,best}^{t}$;

**Step 4:** Return to Step 2 and continue the above process until the desired number of iterations is met.

The corresponding pseudo-code of the PSO which is applied to achieve the optimization energy efficient task scheduling strategy is provided by Algorithm 1.
**Algorithm 1:** The pseudo-code of the PSO used for MR-WSNs.
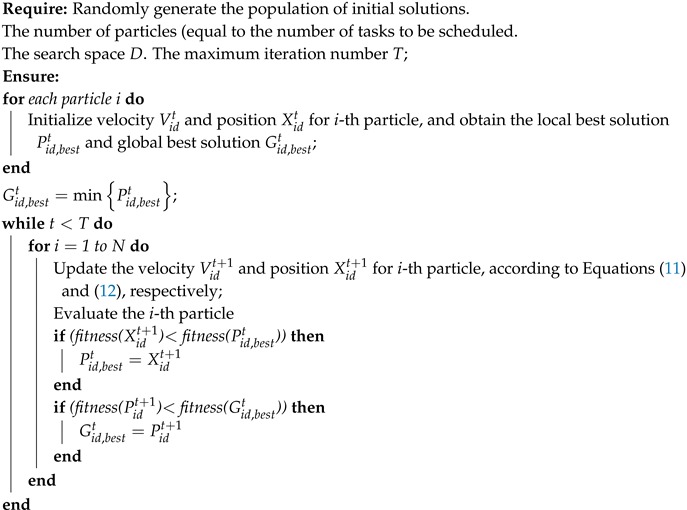


## 5. Performance Evaluation and Analysis

In order to carry out a detailed evaluation of the algorithm’s behavior, some experiments and simulations are deployed. Both the experiments and simulation results are compared with the FIFO algorithm and EMRSA [39] concerning items of average energy consumption, the first dead node, the network lifetime and the average latency. The proposed optimization energy-consumption task scheduling strategy is abbreviated to OETS for convenience. The experiment, the simulations and the proposed strategy based on the PSO algorithm are applied in the remote multi-radio nodes. In detail, the position and velocity correspond to the energy consumption of nodes and the scheduling task of the RF transceiver, respectively. The velocity (scheduling tasks) iteratively updates according to the scheduling results and then updates the position (energy consumption).

### 5.1. Experiment and Analysis

**Figure 2:**
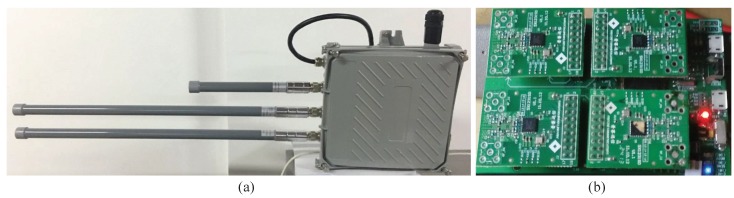
Devices used in experiments; (**a**) multi-radio base station; (**b**) remote multi-radio node.

In the experiment, the testbed consists of forty remote multi-radio nodes and a multi-radio base station. They are randomly deployed on our laboratory test stand. The multi-radio base station and remote multi-radio node are shown in Figure 2.

Table 2 lists some key initialization parameters of the experiments in this section.

**Figure 3:**
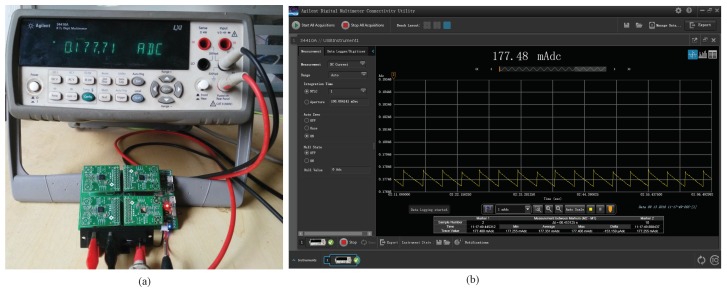
Energy consumption test; (**a**) multi-radio node; (**b**) current consumption recorder.

Figure 3 shows the experimental test hardware and the PC software user-interface, separately. The experiment environment is deployed based on our designed multi-radio hardware (shown in Figure 3a). The designed multi-radio hardware supports four RF transceivers (n=4) at most. The current consumption is recorded by the Agilent Digital Multi-Meter supported PC software (shown in Figure 3b).

The max–min method and the Z-score method are used to normalize the experiment results, aiming at avoiding the confusion that different data units caused. Table 3 lists the normalized value of each algorithm.

**Figure 4:**
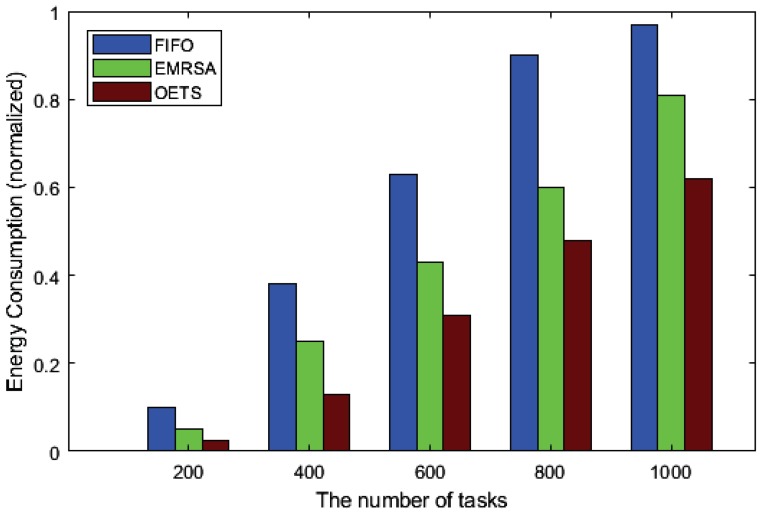
Energy consumption results.

Figure 4 shows the experiment results of the energy consumption performance for FIFO, EMRSA and OETS. In Figure 4, the vertical axis represents the normalized energy consumption. The larger the value, the higher the energy consumption, and the lower the corresponding performance. The horizontal axis represents the number of tasks.

Figure 4 indicates that OETS outperforms both FIFO and EMRSA. In detail, OETS is at most 36.2% lower than FIFO, and 23.1% than EMRSA, under the same number of tasks. The most important reason for FIFO is that the energy consumption factor is totally without consideration. For EMRSA, it is a greedy algorithm and merely considers whether the energy consumption of the current task is optimal. Hence, it may easily fall into the local optimal. Inspired by the PSO algorithm, the proposed OETS not only considers the local best solution, but also takes the global particles best solution into consideration. Then, each particle iteratively updates its solution until obtaining the best one. Hence, the energy consumption of the PSO based task scheduling strategy is the lowest.

### 5.2. Simulation and Analysis

To further evaluate the performances of OETS, some simulations are deployed using Matlab. Table 4 presents some key simulation parameters.

**Figure 5:**
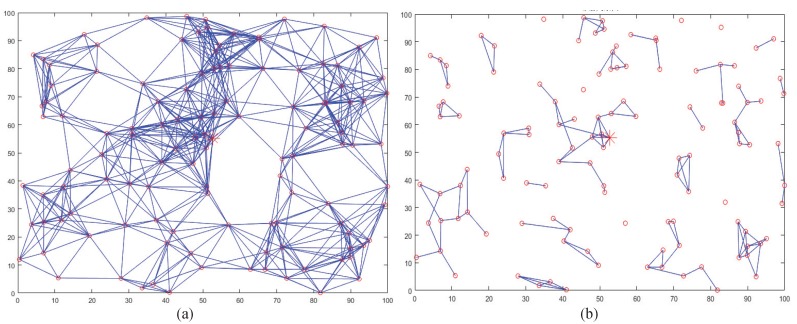
Topologies in simulations; (**a**) the beginning; (**b**) the end.

**Figure 6:**
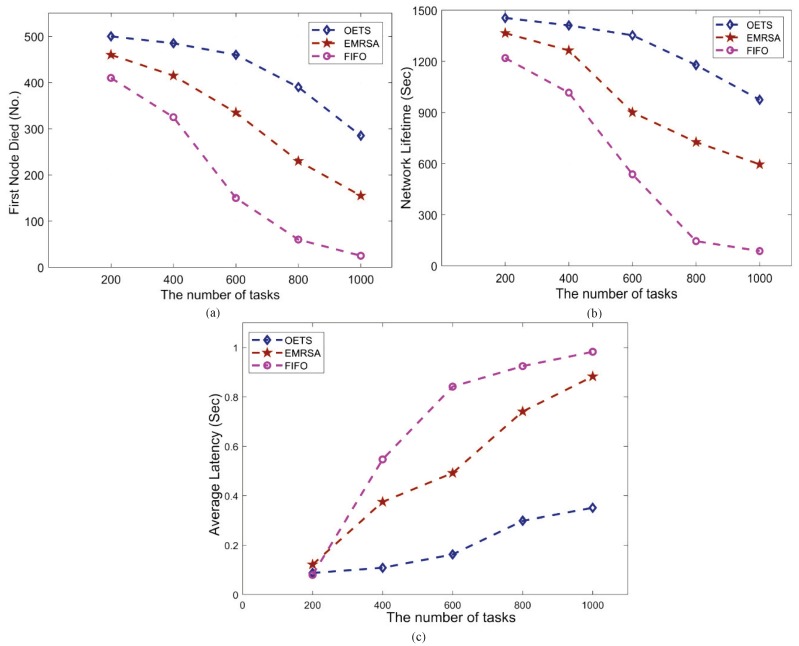
Simulation Results: (**a**) First Node Died, (**b**) Network Lifetime, (**c**) Average Latency.

In the simulation, 120 multi-radio nodes are uniformly deployed from (x = 0, y = 0) to (x = 100, y = 100). The data packet size is 64 bytes. Both the transmit power and idle listening power use the default. Figure 5 shows the network deployment in the beginning (Figure 5a) and the end (Figure 5b). The simulation ends if any one algorithm cannot effectively deliver data. The simulation results, including the first dead node, the network lifetime and the average latency, are shown in Figure 6a–c, respectively.

As shown in Figure 6a, the first dead node is FIFO, then EMRSA and OETS last. Since FIFO does not consider the energy consumption factor, the energy consumption sharply increases along with the increasing of the number of tasks. Hence, the energy is rapidly exhausted and the node quickly dies. For EMRSA, which is greedy algorithm based, it merely optimizes the energy consumption of the current task. The energy consumption stably increases along with the increasing of the number of tasks. Therefore, the node stably exhausts the energy and dies. For our proposed OETS, it considers the global energy consumption to be optimal. The increase of energy consumption is relatively slow along with the number of task increases. Hence, the first node dies last.

Figure 6b shows the network lifetime results of the three algorithms. For FIFO, along with the increase of the number of tasks, the increase of the number of dead nodes is explosive. Therefore, the network rapidly deteriorates and cannot deliver data. For EMRSA, the network lifetime is longer than FIFO, since the nodes naturally die. For OETS, this algorithm effectively decreases the energy consumption and prolongs the lifetime of the node. Hence, the network lifetime is longer than both FIFO and EMRSA.

Figure 6c shows the average latency results of the three strategies. It is clearly observed that the average latency of our proposed strategy outperforms others since the algorithm can dynamically allocate the packets to the current best RF transceiver. For the FIFO algorithm, the latency shapely increases with the increase of the number of tasks, because an increasing number of tasks makes most nodes quickly exhaust their energy and die. That leads to a loss of the effective data link and increases the high latency. For the EMRSA, the latency increases with the increase in the number of tasks, for reasons similar to the FIFO algorithm.

### 5.3. Tasks Extensibility Analysis

**Figure 7:**
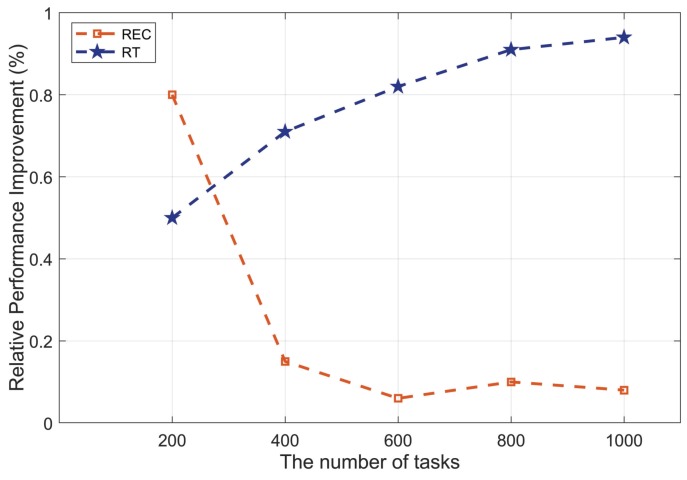
Task extensibility analysis.

In order to further confirm the application scenario of OETS and analyze the task extensibility, the relative performance experiments, including relative energy consumption (REC) and relative time (RT), are conducted. Since the FIFO does not consider the energy consumption factor and the performance of this algorithm is obviously worse than EMRSA and OETS, we do not compare the relative performance with FIFO in this subsection. The relative energy consumption is defined as the proportion of the energy consumption difference between EMRSA and OETS to the energy consumption of EMRSA. The relative time is defined as the proportion of the required calculation time difference between EMRSA and OETS to the required calculation time of EMRSA. Figure 7 shows the results of relative energy consumption and relative time. In Figure 7, the result of relative performance improvement is normalized and represented by the vertical axis. The horizontal axis represents the number of tasks.

As shown in Figure 7, the REC sharply drops among the number of tasks 200~400, while the RT gradually increase in the same interval. The intersection of the two curves lies among the number of tasks 200~400. The rising amplitude of REC exceeds the declining amplitude of RT when the number of tasks lies at the left of the intersection point. This means that the network performances of the OETS algorithm, i.e., the energy consumptions and the required calculation time, are superior to the EMRSA algorithm. When the number of tasks is greater than 400, the situation is converse, namely the rising amplitude of REC is smaller than the declining amplitude of RT. One reason is that the energy consumption rises along with the increasing of the number of tasks. The other reason is that the time required to search for the optimal solution rises as the number of tasks enlarges. Hence, the superiority of the OETS algorithm is not as obvious as before. To sum up the above results, the OETS algorithm is the optimal task scheduling strategy when the number of tasks is no greater than 400.

## 6. Conclusions

In order to fully take advantage of the multi-radio technology in WSNs while reduce the energy consumption, this paper analyzed the two types of energy dissipation processed first, i.e., transmission process and idle listening process, then combined the two types of processes together and obtained the accurate energy consumption model of the multi-radio node. Moreover, the optimization energy consumption problem was converted into a multi-task scheduling problem. Inspired by the PSO algorithm, this paper proposed OETS based on the PSO to search for the best solution of such optimization problems, and then provided the detailed steps of the proposed OETS. Both the experiment and the simulation results were given. Those results showed that the proposed OETS based on the PSO algorithm could effectively improve the energy consumption of a multi-radio node and prolong the network lifetime. Furthermore, the task extensibility of the proposed OETS was better, especially when the number of tasks was less than 400.

In future work, we will deploy more experiments by taking the influence of different topologies into consideration and explore more potential application scenarios. Furthermore, the proposed strategy based on the PSO algorithm will apply in the base station for reducing the energy consumption and prolonging the lifetime of the network. For this application, the position and velocity can respond to the energy consumption of the base station and the remote nodes, respectively.

## Figures and Tables

**Table 1:** Acronyms and parameters used in this paper.

Symbols	Description
*m*	the number of waiting to be scheduled tasks
*n*	the number of RF transceivers
γ	the idle RF resource
$N_{i}$	the *i*-th multi-radio node
$R_{j}$	the *j*-th RF transceiver
$R=\left\{R_{1},R_{2},\cdots ,R_{n}\right\}$	RF resource set
$T=\left\{T_{1},T_{2},\cdots ,T_{m}\right\}$	waiting to be scheduled tasks set
$W=\left\{W_{i,1},W_{i,2},\cdots ,W_{i,l}\right\}$	the *l*-th packets of $T_{i}$
$w_{i}^{R\left(j\right)}=\left\{w_{i,k},w_{i,k+1},\cdots ,w_{i,k+h}\right\}$	the *h* packets allocated to $R_{j}$
$T_{i}$	the *i*-th task
$w_{i,q}$	the *q*-th packet of $w_{i}^{R\left(j\right)}$
$t_{i,q}$	required time for transmitting $w_{i,q}$
$t_{j}$	total time for transmitting $w_{i}^{R\left(j\right)}$
$t_{\max}$	the maximum between $t_{j}$
$S_{i,q}$	energy consumption of transmitting $w_{i,q}$
$P_{trans,j}$	transmission power of $R_{j}$
$S_{j}$	total energy consumption of $w_{i}^{R\left(j\right)}$
$I_{j}$	energy consumed for listening of $R_{j}$
$p_{idle,j}$	idle listening power of $R_{j}$
$E_{j}$	total energy consumption of $R_{j}$
*E*	total energy consumption of $N_{i}$
$E_{current}$	energy consumption of current solution
$E_{new}$	energy consumption of new solution

**Table 2:** Some key initialization parameters.

Parameters	Value	Parameters	Value
No. of multi-radio node	40	Supply current	3A DC
No. of RF transceivers (*n*)	4	Supply voltage	3.3V DC
No. of tasks (*m*)	200–1000	Typical transmit energy consumption	178 mA (17 dB)
No. of packets of each task (*l*)	6	Typical idle energy consumption	2.25 mA (0 dB)

**Table 3:** Results of energy consumption.

No. of Tasks	FIFO	EMRSA	OETS
**200**	0.12	0.052	0.025
**400**	0.375	0.27	0.13
**600**	0.642	0.442	0.31
**800**	0.94	0.613	0.48
**1000**	0.98	0.807	0.62

**Table 4:** Some key simulation parameters.

Parameters	Value	Parameters	Value
Network size	100 × 100 m${}^{2}$	No. of packets (each tasks)	6
No. of nodes	120	Energy model	Battery
No. of RF transceivers (*n*)	4	Data packet size	64 Bytes
Node distribution	Uniform	Transmit power	Default
No. of tasks	200–1000	Idle listening power	Default
